# The Patients' Meat Ration

**Published:** 1917-03-10

**Authors:** 


### SOME WAR PROBLEMS IN FOOD.

### The Patients' Meat Ration.

In facing the problem of the hospital meat ration, managers will undoubtedly be driven to separate patients from staff, and patients on low diet from those on full diet. This elementary classification is still unattempted in far too many institutions in the country. But an estimate of the amount of meat consumed in the entire hospital divided by the entire number of patients and staff would land nowhere. Strictly speaking, those who consume meat are entitled to 2½ lb. of meat a week. Those who do not consume meat should not be reckoned at all, for the aim should be not to consume as much as possible, but to limit the meat-eater's allowance.

It is quite impossible to gauge the average number of patients on diets which exclude meat or poultry without taking the exact number on a given day periodically. Probably the average remains fairly constant in each particular hospital, though varying very widely indeed from one hospital to another. Whether, however, there be an average of 3 per cent. or of 40 per cent. not receiving meat in any form, or either broth or beef-tea, this number should be excluded from the total in reckoning out the meat ration. For the remainder an allowance of 5.7 oz. of meat a day is available, including bone. How does this compare with the amount prescribed in the diet tables?

The usual allowance of meat for patients on full diet is 6 oz. for men and 4 oz. for women, cooked, without bone. It is not usual to weigh the portions, so that this is a rough average. At Guy's Hospital, where the quantities were carefully computed, this allowance of cooked meat was found to work out in practice at 8⅔ oz. of meat as received from the butcher uncooked.

In considering the effect of the war ration on the patients' diets we are not oblivious of the fact that the sick are dispensed from rigid adherence to the rules imposed on the healthy. But we propose to show that managers may work very closely indeed to the war ration and yet inflict no hardship on those on full diet. If there be waste in feeding the patients it ought to be ended.

The extent to which meat loses weight and furnishes waste in roasting is very imperfectly understood among those responsible for dieting large numbers. In "Hospital Expenditure—The Commissariat," a table is given which shows the result of baking two fairly large legs of mutton, English and New Zealand respectively, and the amount of diets which were carved from them. The English leg (8 oz. heavier than the foreign one) lost 2 lb. 7 oz. in the cooking, against 2 lb. 1 oz. for the New Zealand leg. The weight of slices suitable for diets was 3 lb. 4 oz. and 3 lb. respectively, and the weight of bone, gristle, etc., left on the dish after every scrap of meat had been cut off amounted in each case to 2 lb. 5 oz. Careful housekeepers would do well to test these experiments for themselves. We fear they will reach the conclusion that very little more than two-thirds of the meat as it is delivered uncooked from the butcher reaches the mouths of the patients after it has been roasted.

The problem, then, becomes considerably more difficult than it appears at first sight. Not an average per head of 2½ lb. of meat, but an average per head of about 1¾ lb. is what the patients may actually consume according to the war ration. This means a weekly weight of roast meat without bone of about 28 oz., or a daily average of 4 oz. In other words, the war ration of the male patients amounts to the allowance of meat hitherto deemed sufficient for the weaker sex.

This calculation is made on the assumption that the meat is served to the patients roasted or baked. But undoubtedly this is a wasteful method of serving meat under present circumstances. We commend to housekeepers a very simple experiment. Let two joints of a given weight, say of boned ribs of beef, be cooked (*a*) by baking; (*b*) by braising or stewing. Then let each joint be weighed with all the gravy and appurtenances thereof, and its weight compared with that prior to cooking. A clear demonstration will be provided of the extent to which meat wastes by the removal of moisture in baking and gains by the addition and conservation of moisture under any form of conservative cookery. Moreover, in baking or roasting a great deal of meat is wasted on the outer edges, which dry up and become too hard, at any rate for invalids. When meat is stewed or braised every particle can be devoured, and the valuable juices which are apt to dry on the sides of the baking-tin are saved to the last drop. The simplest form, then, of war economy is to substitute stews and braised meat for the roast joint, and use up the bones for soup. By this means at least one ounce a day more of good meat may be secured for each patient out of his war ration, for the satisfying of appetite.

For "The Nursing Problem in Ireland," see p. 467.
For Royal Red Cross Honours, see pp. 468, vi..

